# Association of polymorphisms at the microRNA binding site of the caprine *KITLG* 3′-UTR with litter size

**DOI:** 10.1038/srep25691

**Published:** 2016-05-11

**Authors:** Xiaopeng An, Yuxuan Song, Shuhai Bu, Haidong Ma, Kexin Gao, Jinxing Hou, Shan Wang, Zhang Lei, Binyun Cao

**Affiliations:** 1 College of Animal Science and Technology, Northwest A&F University, Yangling, Shaanxi 712100, P.R. China; 2 College of Life Science, Northwest A&F University, Yangling, Shaanxi 712100, P.R. China

## Abstract

This study identified three novel single nucleotide polymorphisms (SNPs) (c.1389C > T, c.1457A > C and c.1520G > A) in the caprine *KITLG* 3′-UTR through DNA sequencing. The three SNP loci were closely linked in Guanzhong dairy (GD) goats. Two alleles of the c.1457A > C SNP introduced two miRNA sites (chi-miR-204-5p and chi-miR-211). Individuals with combined genotype TT-CC-AA had a higher litter size compared with those with combined genotypes CC-AA-GG, TC-CC-GA and CC-AC-GG (*P* < 0.05). Luciferase assays showed that chi-miR-204-5p and chi-miR-211 suppressed luciferase expression in the presence of allele 1457A compared with negative control (NC) and allele 1457C (*P* < 0.05). Western blot revealed that KITLG significantly decreased in the granulosa cells (GCs) of genotype AA compared with that in the GCs of genotype CC and NC (*P* < 0.05). The *KITLG* mRNA levels of the CC-AA-GG carriers significantly decreased compared with those of the TT-CC-AA, TC-CC-GA and CC-AC-GG carriers. In addition, cell proliferation was reduced in haplotype C-A-G GCs compared with that in haplotype T-C-A GCs. These results suggest that SNPs c.1389C > T, c.1457A > C and c.1520G > A account for differences in the litter size of GD goats because chi-miR-204-5p and chi-miR-211 could change the expression levels of the *KITLG* gene and reduce GC proliferation.

KIT ligand (KITLG), also known as stem cell factor, steel factor and mast cell growth factor, has multiple biological functions during the development of goat, rats and humans by triggering its receptor tyrosine kinase (KIT)^1^,^2^,^3^. The caprine *KITLG* gene contains ten exons and nine introns^4^. This gene participates in the survival and proliferation of granulosa cells (GCs), in the recruitment of theca cells from ovary stroma and in the regulation of steroidogenesis^5^. In the ovary, *KITLG* mRNA is expressed in the GCs of many species; it can be expressed as either a membrane-bound (KL-1) or a soluble protein (KL-2) depending on the mRNA processing after transcription^6^. The mRNA expression of *KITLG* remains high in early antral follicles^7^ but decreases as follicular growth progresses towards the late antral stage without any significant alteration in the ratio between KL-1 and KL-2^8^. Essentially similar results have been reported for sheep follicles^9^. The study of animal models has revealed that the interaction of GC-derived KITLG with oocyte and theca cell-derived KIT is important for multiple aspects of oocyte and follicle development, including primordial germ cell establishment within the ovary, primordial follicle activation, oocyte survival and growth, GC proliferation, theca cell recruitment and meiotic arrest maintenance^10^. A blockage of KIT function affects the onset of primordial follicle development, primary follicle growth, follicular fluid formation and preovulatory follicle development^11^. These findings indicate that the *KITLG* gene may be an excellent candidate for reproductive traits in humans and livestock.

MicroRNAs (miRNAs) are small non-coding RNA molecules (each containing about 22 nucleotides) that post-transcriptionally regulate the expression of their target genes via either translational repression or mRNA degradation by binding to the complementary seed sites within the 3′-untranslated region (3′-UTR) of the target mRNA^12^,^13^. MiRNAs modulate diverse biological processes, including embryonic development, cell proliferation and differentiation, apoptosis, fat metabolism, atherosclerosis and oncogenesis^14^,^15^. SNPs that affect miRNA binding of target genes are called miR-SNPs^16^. Brodersen and Voinnet^17^ showed that SNPs in miRNA binding sites can affect miRNA-induced genetic repression. Tan *et al*.^18^ indicated that the rs17592236 (C → T) polymorphism could decrease hepato cellular carcinoma hereditary susceptibility by modulating the binding affinity of miR-137 to the 3′-UTR in *FOXO1* mRNA. Zhang *et al*.^19^ provided the first indication of g.442A > G-mediated and g.528G > A-mediated translational suppression in which SNPs alter the binding of bta-miR-204 and bta-miR-532 to the 3′-UTR of TNP1; the mediated translational suppression could be involved in the regulation of TNP1 expression and may influence the morphological characteristics of Chinese Holstein bull sperm. Liu *et al*.^20^ reported that the human SNP rs3735590 C → T influences miR-616 binding to the *PON1* gene, thereby increasing the risk of ischemic stroke and carotid atherosclerosis. Clop *et al*.^21^ demonstrated that the *GDF8* allele of Texel sheep is characterised by a G → A transition in the 3′-UTR that creates a target site for miR-1 and miR-206, which are highly expressed in skeletal muscle; consequently, translational inhibition of the myostatin gene occurs and contributes to muscular hypertrophy in Texel sheep. Given the regulatory role of miRNAs in gene expression, miR-SNPs may function as promising markers for reproductive traits.

The present study aims to elucidate the potential molecular mechanism which regulates the caprine *KITLG* gene expression and the role of *KITLG* gene in litter size in GD goats. The following parameters were investigated: 1) the association of combined genotypes of the *KITLG* gene with litter size in the GD goats; (2) the potential target miRNAs of the *KITLG* gene; 3) the effect of target miRNAs on *KITLG* gene expression by the functional SNP of the caprine *KITLG* gene; and 4) the relationships among functional SNP, target miRNAs and litter size in GD goats.

## Results

### SNP identification, genotyping and association analysis

**Figure 1:**
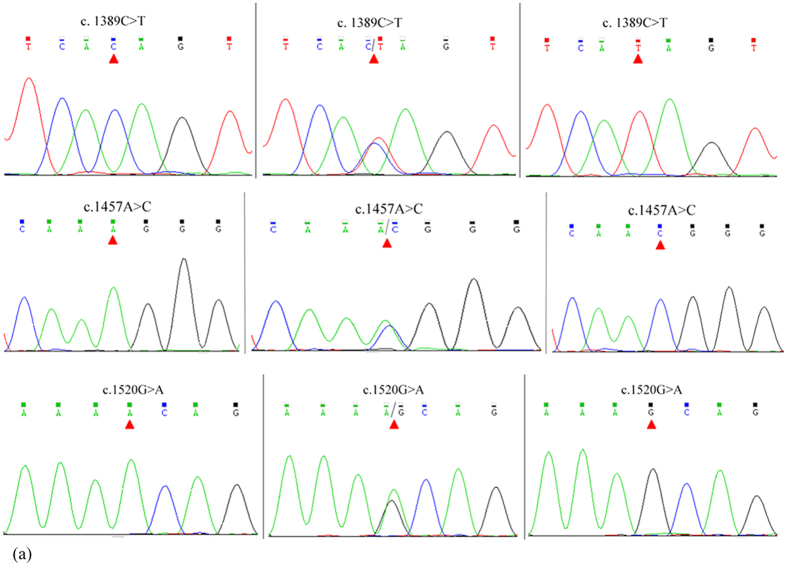
Sequencing chromatograms of different genotypes at the c.1389C > T, c.1457A > C and c.1520G > A loci.

Three SNPs (c.1389C > T, c.1457A > C and c.1520G > A) were identified in the caprine *KITLG*3′-UTR by DNA sequencing (Fig. 1). The mutant sequence of *KITLG* 3′-UTR was submitted to NCBI (GenBank Accession No. KR869087). Two alleles of the c.1457A > C SNP introduced two miRNA sites (Figure S1). The SNPs c.1389C > T and c.1520G > A had no effect on miRNA sites. The polymorphism information contents (PICs) were 0.35 and 0.37 at the c.1389C > T, c.1457A > C and c.1520G > A loci (Table S3). The c.1389C > T, c.1457A > C and c.1520G > A loci were in Hardy–Weinberg disequilibrium (*P* < 0.05). The genotypic distribution and allele frequencies of three SNPs are shown in Table S3. The linkage disequilibrium was estimated in GD goats to reveal the linkage relationships among the three SNPs (Figure S2^22^,^23^). The c.1389C > T, c.1457A > C and c.1520G > A loci were closely linked in GD goats (Figure S2). In consideration of the close linkage of the three loci, the association of the combined genotypes with litter size was analysed. Results showed that individuals with combined genotype TT-CC-AA had the highest litter size compared with those with combined genotype**s** CC-AA-GG, TC-CC-GA and CC-AC-GG (*P* < 0.05, Table 1).

### SNP c.1457A > C of *KITLG* 3′-UTR affects KITLG expression and cell proliferation

**Figure 2:**
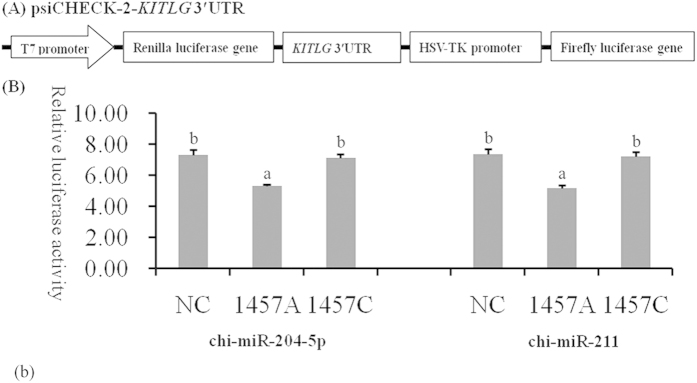
Characterisation and functional analysis of *KITLG* 3′-UTR. (**A**) Double fluorescein enzyme expression vector linked with *KITLG* 3′-UTR. (**B**) Granulosa cells seeded on 24-well plates were transiently co-transfected with psiCHECK-2-*KITLG* 3′-UTR and miRNA mimics (chi-miR-204-5p and chi-miR-211) or stable negative control (NC). Luciferase activity was measured after 36 h of transfection. *Renilla* luciferase was normalisedwith firefly luciferase activity, and the mean activities ± standard deviation from three independent experiments are shown. The different small letters represent significant difference at the 5% level.

**Figure 3:**
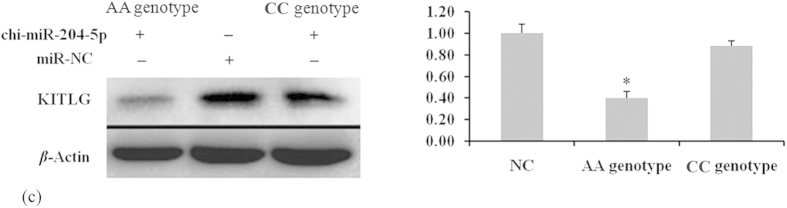
KITLG expression is suppressed by chi-miR-204-5p in granulosa cells. (**A**) Western blot analysis of KITLG expression in CC and AA genotype granulosa cells transfected with either chi-miR-204-5p or negative control (NC). The expression of β-actin was used as a loading control. (**B**) Densitometric quantification of three Western blot results for KITLG relative to β-actin levels. Data represent the means ± standard deviation of three independent experiments, where an asterisk denotes significance between CC and AA genotype granulosa cells at *P *< 0.05. Note: The 16 bases inthe 5′region sequence of chi-miR-204-5p are the same as that of chi-miR-211. Thus, chi-miR-204-5p was used in Westernblot analysis.

**Figure 4:**
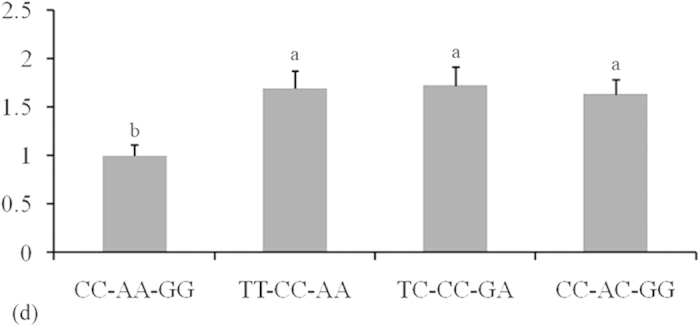
Comparison of mRNA expression levels of caprine *KITLG* in granulosa cells among four combined genotypes. The number of animals of combined genotypes CC-AA-GG, TT-CC-AA, TC-CC-GA and CC-AC-GG were 9, 10, 5 and 6, respectively. The fold change was normalised against the *β-actin* and *GAPDH* genes. The different small letters represent significant difference at the 5% level.

A luciferase reporter assay was used to understand the functional significance of the c.1457A > C substitution. As shown in Fig. 2B, chi-miR-204-5p and chi-miR-211 suppressed luciferase expression in the presence of allele 1457A compared with NC and allele 1457C (*P* < 0.05). GCs of different genotypes were transfected with chi-miR-204-5p mimics to determine whether chi-miR-204-5p affects the protein level of KITLG. In consideration that the 16 bases in the 5′ region sequences of chi-miR-204-5p and chi-miR-211 are the same, chi-miR-204-5p was used in Western blot analysis. Western blot analysis revealed that KITLG significantly decreased in the GCs of genotype AA compared with that in the GCs of NC and genotype CC (*P* < 0.05, Fig. 3). These results suggest that individuals with genotype AA could have lower KITLG protein expression levels than those with genotype CC. The mRNA expression of *KITLG* was assessed in 30 dairy goats with different combined genotypes for the c.1389C > T, c.1457A > C and c.1520G > A loci. Individuals with combined genotype CC-AA-GG had lower *KITLG* mRNA expression levels compared with those with combined genotypes TT-CC-AA, TC-CC-GA and CC-AC-GG (Fig. 4).

**Figure 5:**
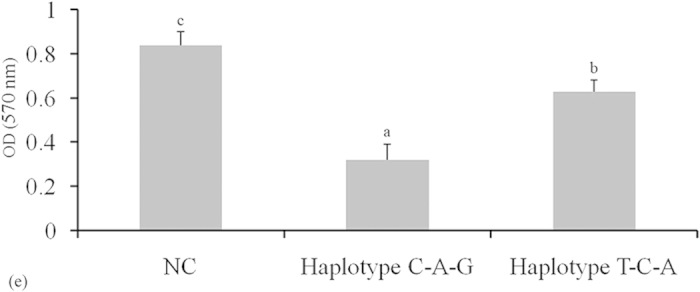
Effect of chi-miR-204-5p on granulosa cell viability. The cells were cultured in chi-miR-204-5p mimics or stable negative control (NC) for 36 h. Granulosa cell viability was measured using the MTT assay (mean ± standard deviation, n = 3 independent culture experiments). Small letters represent significant difference at the 5% level.

Cell proliferation ability was analysed using 3-(4,5-dimethylthiazol-2-yl)-2, 5-diphenyl-2H-tetrazolium bromide (MTT) at 36 h after transfection in GCs to evaluate the effects of chi-miR-204-5p on cell proliferation. Cell proliferation was reduced in haplotype C-A-G GCs compared with that in haplotype T-C-A GCs (*P* < 0.05, Fig. 5).

## Discussion

In the present study, the three SNPs showed Hardy–Weinberg disequilibrium in GD goats (*P *< 0.001). Thus, the population analysed may be subject to evolutionary forces, such as selection, mutation or migration. Genome-wide bioinformatics analysis predicted that approximately 64% of transcribed SNPs as target SNPs of miRNAs can modify (increase/decrease) the binding energy of putative miRNA–mRNA duplexes by >90%^24^. MicroRNA-related SNPs are functional SNPs which may impair the regulatory function of miRNA. The 3′-UTR SNPs may be within or at the vicinity of the miRNA binding site; these polymorphisms may interfere with miRNA function, lead to differential gene expression and ultimately affect the animal phenotype^25^. Follicle development is a complex multistep developmental process which involves continuous cell proliferation and differentiation. Differentiation is dependent on sequential gene expression. MiRNAs are involved in the development of follicles and are abundant in caprine ovaries^26^. In follicle development, the miRNA–argonaute complex interacts with the 3′-UTR of the target mRNAs by the complementary binding of an miRNA to an mRNA. This binding blocks translation initiation, induces endonucleolytic cleavage of a target mRNA or both^27^. The seed region of miRNAs (nucleotides 2–8 of the 5′-end) is important in mRNA-targeting efficacy^28^. In particular, miRNAs require almost perfect complementarity at the seed sites to bind and to reduce the protein levels of targets^17^,^29^. In the present study, the c.1457A > C mutation occurred in the seed region of chi-miR-204-5p binding sites and increased the protein levels of KITLG because the base C reduced the binding affinity of chi-miR-204-5p and the 3′-UTR of the *KITLG* gene.

The SNP g.1536C > T in the *TNP2* 3′-UTR, which alters the binding of *TNP2* with bta-miR-154, is associated with the semen quality traits of Chinese Holstein bulls^30^. An *et al*.^31^ suggested that the SNP g.173057T > C of *PRLR* 3′-UTR, which influences the binding activity of bta-miR-302a and 3′-UTR, significantly influences litter size of goats. In the present study, association analysis showed that combined genotype TT-CC-AA could be used to select individuals with large litter sizes. The results of the luciferase assay are consistent with those of *in silico* analysis; this consistency suggests that functional interaction occurs between chi-miR-204-5p or chi-miR-211 and the *KITLG* 3′-UTR. The *KITLG* mRNA levels were further examined in GD goats with different combined genotype**s**. The *KITLG* mRNA levels of the CC-AA-GG carriers significantly decreased compared with those of the TT-CC-AA, TC-CC-GA and CC-AC-GG carriers. In addition, cell proliferation was reduced in haplotype C-A-G GCs compared with that in haplotype T-C-A GCs. Therefore, the SNPs c.1389C > T, c.1457A > C and c.1520G > A may account for the different litter sizes of GD goats because chi-miR-204-5p and chi-miR-211 could change the expression level of the *KITLG* gene and reduce GC proliferation. The combined genotype TT-CC-AA can be used as a molecular marker in dairy goat breeding programs.

In conclusion, the SNP c.1457A > C in the caprine *KITLG* gene is a functional SNP in chi-miR-204-5p and chi-miR-211 target sites. The SNPs c.1389C > T, c.1457A > C and c.1520G > A are closely linked in GD goats and are also possibly involved in follicle development. This study proposes that the SNPs in the 3′-UTR of *KITLG* gene may help select the litter size of dairy goats in the dairy industry. Furthermore, these findings could help us understand the regulatory mechanisms of biological functions and the expression of the *KITLG* gene in dairy goats.

## Materials and methods

### Ethics statement

All animals were maintained in accordance with the No. 5 proclamation of the Ministry of Agriculture, China. Sample collection was approved by the Institutional Animal Care and Use Ethics Committee of Northwest A&F University and performed in accordance with the “Guidelines for Experimental Animals” of the Ministry of Science and Technology (Beijing, China). The Institutional Animal Care and Use Ethics Committee of Northwest A&F University approved this study.

### Animals and genomic DNA isolation

Blood samples were obtained from 385 Guanzhong dairy goats reared in Longxian County of Shaanxi Province. All diets were based on alfalfa, corn silage and a combination of concentrates, such as corn, soya meal and bone meal. Health, fertility and production records were maintained by the dairy producers and veterinarians. The litter size from the first to fourth parity was obtained from production records. Blood samples (5 mL) were collected aseptically from the jugular vein of each individual and stored in a tube containing anticoagulant ACD (citric acid : sodium citrate : dextrose = 10:27:38). All samples were delivered to the laboratory in an ice box. The genomic DNA was extracted from white blood cells by using a standard phenol–chloroform extraction protocol.

### SNP investigation and genotyping

Bovine and caprine *KITLG* genes (GenBank Accession Nos. AC_000162 and NM_001285670) were used to design five pairs of primers to amplify the 3′-UTR of the caprine *KITLG* gene. Their optimal annealing temperatures are shown in Table S1. Subsequently, the samples were screened to identify the SNPs of the *KITLG* gene through pooled DNA sequencing^32^. The DNA pool of dairy goats was created by mixing 5 μL of DNA (100 ng/μL) from 385 Guanzhong dairy goats. The pooled DNA was used as a template to amplify the 3′-UTR of the caprine *KITLG* gene. A PCR Master Mix (2×) Kit (Thermo Scientific, NY, USA) was used for PCR amplification. The 25 μL PCR reactions contained 50 ng of pooled genomic DNA, 12.5 μL of 2× reaction mix (including 500 μM of each dNTP; 20 mM Tris–HCl, pH 9; 100 mM KCl; 3 mM MgCl_2_), 0.5 μM of each primer and 0.5 U of *Taq* DNA polymerase (Thermo Scientific, NY, USA). The cycling protocol involved initial denaturation at 95 °C for 5 min;35 cycles of denaturation at 94 °C for 30 s, annealing at 50–53 °C for 30 s and extension at 72 °C for 35 s; and a final extension at 72 °C for 10 min. Ten PCR products amplified with pooled DNA as a template were sent to the Beijing Genomics Institute (Beijing, China) for Sanger sequencing in both directions. SNP discovery was conducted using the Chromas version 2.31 and DNAstar version 7.0 software. SNP nomenclature and numbering followed the recommendations of http://www.hgvs.org/mutnomen/recs-DNA.html#number. If SNPs were found in the 3′-UTR of the caprine *KITLG* gene, each sample was amplified independently. Then, the PCR products of each sample were sequenced independently to detect different genotypes by sequencing.

### Statistical analysis

The allele frequencies, heterozygosity (He) and PIC were calculated using POPGENE (version 1.31, http://www.ualberta.ca/~fyeh/popgene _download.html). Association analysis between combined genotypes and litter size was performed using repeated measures in the general linear model procedure of SPSS version 16.0 statistical software (http://en.softonic.com/s/spss-16-software). The mixed linear model was described by *Y*_ijkl_ = *μ* + *G*_i_ + *P*_*j*_ + *S*_k_ + *E*_ijkl_, where *Y*_ijkl_ is the trait measured in each of the ijkl^th^ female goat, *μ* is the overall population mean, *G*_i_ is the fixed effect associated with the i^th^ genotype (TT-CC-AA, CC-AA-GG, TC-CC-GA or CC-AC-GG), *P*_*j*_ is the fixed effect associated with the j^th^ parity (from first to fourth parity), *S*_k_ is the fixed effect associated with the k^th^ sire (sire number: 7, 10, 12, 18, 23, 45, 75, 180, 239, 278, 290 and 301) and *E*_ijkl_ is the random error. Multiple comparisons of the means for different genotypes were performed via Fisher’s least significant difference test by SPSS version 16.0. The effects associated with the farm, birth year and birth season were not included in the linear model because preliminary statistical analyses indicated that these effects did not significantly influence the variability of traits in the analysed populations.

### Bioinformatics analysis of *KITLG*3′-UTR

Bioinformatics analysis was used to predict the effects of mutations in the 3′-UTR of the *KITLG* gene on the miRNA binding sites. The analysis was performed with five bioinformatics tools, namely,

MicroInspector (http://bioinfo1.uni-plovdiv.bg/cgi-bin/microinspector/),

TargetScan (http://www.targetscan.org/),

miRWalk (http://www.umm.uni-heidelberg.de/apps/zmf/mirwalk/index.html), RNAhybrid (http://bibiserv.techfak.uni-bielefeld.de/rnahybrid/submission.html) and Segal Lab (http://genie.weizmann.ac.il/pubs/mir07/mir07_prediction.html). According to a minimal free energy hybridisation of RNA sequence and miRNA (ΔΔG value), these five tools were used to predict miRNA binding sites. The lower (more negative) the value of the energetic score ΔΔG, the stronger the binding of the microRNA to the given site is expected to be. The combination of these approaches was considered to reduce the possibility of false positives.

### Cell collection

Goat GCs were collected from the ovaries of Guanzhong dairy goat by using the follicle isolation method as previously described^33^. Viable GCs were cultured in 24-well plates with 0.5 mL of DMEM/F-12 supplemented with 10% foetal bovine serum, 100 U/mL penicillin and 100 μg/mL streptomycin. The cells were cultured in a 5% CO_2_ humidified incubator at 37 °C. Cell transfection was performed when the cells reached a confluence of 80%.

### Plasmid construction and luciferase reporter gene assay

Fragments of the *KITLG* 3′-UTR with genotypes AA and CC of the c.1457A > C locus were amplified through PCR to construct the luciferase reporter plasmids containing the *KITLG* 3′-UTR. The PCR products of genotypes AA and CC were isolated and separated through agarose gel electrophoresis. These products were linked to a pMD19-T vector with a TA Cloning Kit (Invitrogen, CA, USA). The recombinant pMD19-T vectors with different genotypes were digested by the *Xho*I and *Not*I endonuclease enzymes. Finally, the digested products with different genotypes were inserted between the *Renilla* and firefly luciferase genes in a psiCHECK-2 vector (Promega, WI, USA).The plasmids containing genotypes AA and CCwere obtained (Fig. 2A) and then confirmed by sequencing. For the luciferase reporter assay, GCs were placed in 24-well plates (1 × 10^5^ cells per well) and then co-transfected with psiCHECK-2 vectors containing the 3′-UTR genotypes AA and CC. The mimics of miRNAs (chi-miR-211 and chi-miR-204-5p) and their negative controls (GenePharma, Shanghai, China) were co-transfected with the reporter plasmids to a final concentration of 20 nmol/μL. For GCs, the *Renilla* luciferase activity in lysates was measured 36 h after transfection with the Dual-Luciferase Reporter Assay System (Promega, WI, USA). The results were normalised against firefly luciferase activity. Assays were performed in accordance with the manufacturer’s suggestions. Each experiment was independently performed three times, and each sample was evaluated in triplicate. The Mann–Whitney test was used to compare data, and a two-sided *P* value of 0.05 was considered significant.

### RNA isolation and real-time quantitative PCR (RT-qPCR)

To detect the correlation between the expression levels of *KITLG* mRNA and SNPs *in vivo*, ovarian GCs were directly isolated from 30 ovarian tissues with different genotypes (CC-AA-GG, *n* = 9; TT-CC-AA, *n* = 10; TC-CC-GA, *n* = 5 and CC-AC-GG, *n* = 6) for RNA extraction. Total RNA was extracted from these samples using TRIzol Reagent (Invitrogen, Carlsbad, USA). The extracted RNA was homogenised for RT-qPCR. The quantity and integrity of each RNA sample were assessed with an Agilent 2100 Bioanalyser (Agilent Technologies, USA). A total of 30 RNA samples, which included the genotypes CC-AA-GG, TT-CC-AA, TC-CC-GA and CC-AC-GG, were subjected to reverse transcription using a cDNA High Capacity Kit (Invitrogen, Carlsbad, USA). RT-qPCR analysis with the SYBR Green PCR Master Mix (Takara, Dalian, China) was performed on a CFX Connect Real-time PCR Detection System (Bio-Rad, CA, USA). The relative expression levels of objective mRNAs were calculated using the ∆∆Ct method. The primers of the *KITLG, β-actin* and *GAPDH* genes are described in Table S2. The fold changes were normalised by the expression levels of *β-actin* and *GAPDH*, and each assay was performed in triplicate^34^.

### MTT assay

The GCs’ different genotypes were seeded in 96-well culture plates at a density of 5 × 10^4^/well. After 24 h, the cells were treated with chi-miR-204-5p mimic for 36 h. At the end of culture, 50 μL of MTT (Amresco) was added to each well, including controls. After 4 h, the liquid in the wells was drawn out, 150 μL of DMSO was added to each well (including controls), and then the cells were incubated for 10 min at 37 °C. The OD at 570 nm was determined using an Epoch microplate spectrophotometer.

## Additional Information

**How to cite this article**: An, X. *et al*. Association of polymorphisms at the microRNA binding site of the caprine *KITLG* 3′-UTR with litter size. *Sci. Rep.* **6**, 25691; doi: 10.1038/srep25691 (2016).

## Supplementary Material

Supplementary Information

Supplementary Information

## Figures and Tables

**Table 1:** Combined effect of three loci on litter size (means ± standard errors) in GD goats.

Combined genotype	Number	Litter size
TT-CC-AA	112	2.01 ± 0.03^a^
CC-AA-GG	150	1.72 ± 0.02^b^
TC-CC-GA	46	1.70 ± 0.04^b^
CC-AC-GG	77	1.69 ± 0.03^b^

Note: Values with different superscripts differ significantly at *P* < 0.05.
