# Supplementary material for: Association of polymorphisms at the microRNA binding site of the caprine KITLG 3′-UTR with litter size
Source: Sci Rep. 2016 May 11;6:25691. doi: 10.1038/srep25691 (PMC4863368; doi:10.1038/srep25691)
Supplement: Supplementary Information [file srep25691-s1.pdf]

Association of polymorphisms at the microRNA binding site of the caprine *KITLG* 3′-UTR with litter size

Xiaopeng An[1a], Yuxuan Song[1a], Shuhai Bu[1b], Haidong Ma[a], Kexin Gao[a], Jinxing Hou[a], Shan Wang[a], Zhang Lei[a], Binyun Cao [a]*

Supplementary tables

Table S1 Primer information of *KITLG* gene for detecting SNP and cloning 3′UTR

Primer	Sequence (5'→3')	Objective	GenBank accession No. NM_001285670	Product size (bp)	Ta (^{o}C)
KF1	GCCTCCAGAAGCATCTAAC	Screening	1322	275	51
KR1	CAAGTTCAGACCCACATCC	polymorphism	1596		
KF2	CTCGAGGCCTCCAGAAGCATCTAAC	Cloning	1322	289	53
KR2	GCGGCCGCCAAGTTCAGACCCACATCC	3′UTR	1596		
KF3	TAGCATCATCCACTGTTCAT	Screening	1493	298	50
KR3	ATGTCCTCCTTGGCAGAT	polymorphism	1790		
KF4	GACTTAGGAGGAATCACTGA	Screening	1908	386	50
KR4	AACGCAATGTCCATCTTG	polymorphism	2293		
KF5	TGAGTGAATTTGGCAGGAA	Screening	2253	250	51
KR5	GGTGGTAGGATATGGCAATA	polymorphism	2502		
KF6	CCTTCAATAGAGTGACACAG	Screening	2512	344	50
KR6	CAGGTCCTTACAGCCATTA	polymorphism	2855		

Note: Ta = annealing temperature. The underlined bases in KF2/KR2 represent *Xho*I and *Not*I endonuclease enzyme loci.

Table S2 Primer information for real-time quantitative PCR

GenBank accession No.	Gene	Sequence (5'→3')	Region	Product size (bp)	Ta (^oC)
NM_001285670	*KITLG*	F: GTTGCAGCCAGCTCCCTTAG	722	95	60
		R: GCTACGGCTGCCCATTGTAG	816		
AF481159	*β-actin*	F: CCAAAGCCAACCGTGAGAA	80	101	60
		R: AGAGGCGTACAGGGACAGCA	180		
AJ431207	*GAPDH*	F: CACCCTCAAGATTGTCAGC	324	106	60
		R: CAGTGGTCATAAGTCCCTCC	430		

Table S3 Genotypic distribution of three loci in the caprine *KITLG* gene

Locus			Number of animals
c. 1389C>T	Genotype	CC	227
		CT	46
		TT	112
	Allele	C	0.65
		T	0.35
	He		0.12
	PIC		0.35
	Equilibrium χ^2 test		
c.1457A>C	Genotype	AA	150
		AC	77
		CC	158
	Allele	A	0.49
		C	0.51
	He		0.20
	PIC		0.37
	Equilibrium χ^2 test		*P*<0.001
c.1520G>A	Genotype	GG	227
		GA	46
		AA	112
	Allele	G	0.65
		A	0.35
	He		0.12
	PIC		0.35
	Equilibrium χ^2 test		*P*<0.001
