# Supplementary material for: Association of polymorphisms at the microRNA binding site of the caprine KITLG 3′-UTR with litter size
Source: Sci Rep. 2016 May 11;6:25691. doi: 10.1038/srep25691 (PMC4863368; doi:10.1038/srep25691)
Supplement: Supplementary Information [file srep25691-s2.pdf]

Association of polymorphisms at the microRNA binding site of the caprine *KITLG* 3′-UTR with litter size

Xiaopeng An[1a], Yuxuan Song[1a], Shuhai Bu[1b], Haidong Ma[a], Kexin Gao[a], Jinxing Hou[a], Shan Wang[a], Zhang Lei[a], Binyun Cao [a]*

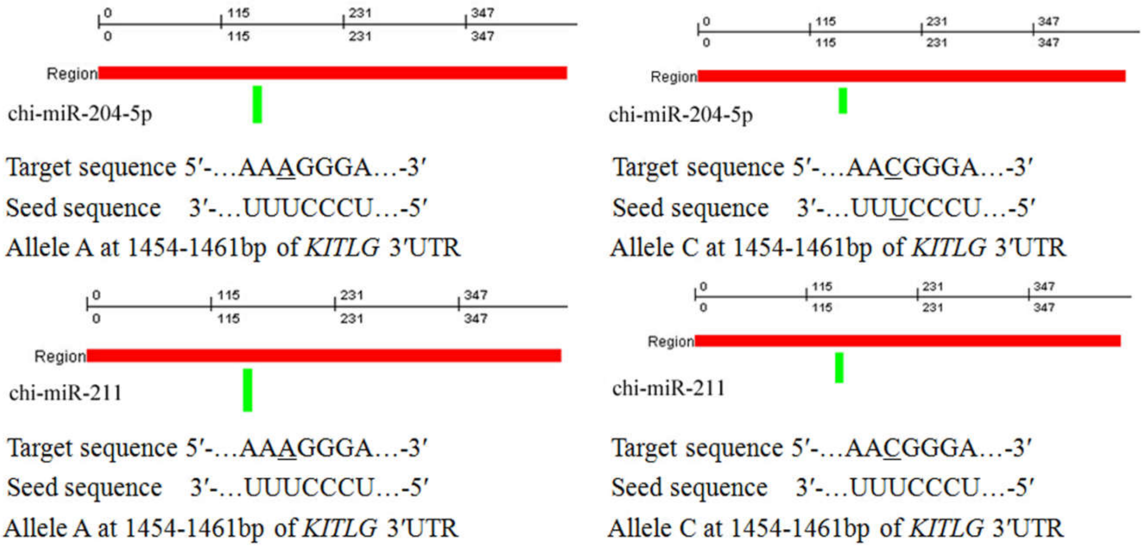

Figure S1 Effects of SNP c.1457A>C on miRNA binding sites in the caprine *KITLG* 3′UTR. The green boxes represent potential miRNA target sites. The underline indicates the position of base mutation

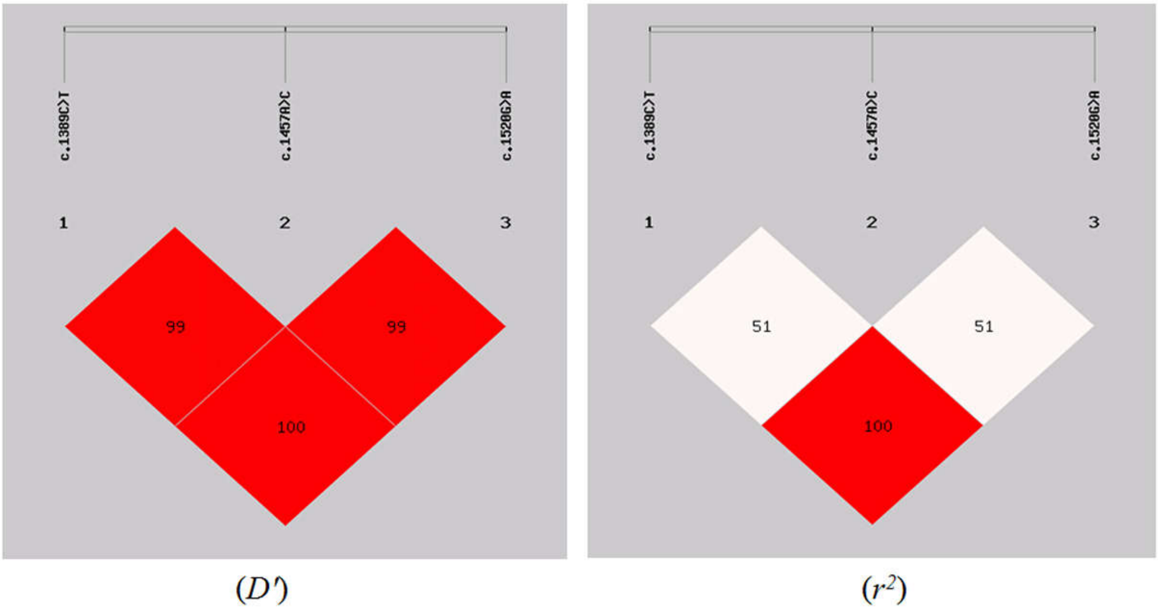

Figure S2 Linkage disequilibrium (LD) analysis of three SNP loci in the GD goats. Numbers in each cell represent pairwise D' and r^2 values (%).

Note: When $r^2 > 0.33$ and $D' > 0.5$, the linkage disequilibrium was considered strong [22, 23].
